# Use of the Uteroglobin Platform for the Expression of a Bivalent Antibody against Oncofetal Fibronectin in *Escherichia coli*

**DOI:** 10.1371/journal.pone.0082878

**Published:** 2013-12-19

**Authors:** Elisa Ventura, Mattia Riondato, Gianmario Sambuceti, Annalisa Salis, Gianluca Damonte, Cinzia Cordazzo, Hüseyin Besir, Vito Pistoia, Luciano Zardi

**Affiliations:** 1 Laboratory of Oncology, G. Gaslini Institute, Genova, Italy; 2 Department of Health Science, Nuclear Medicine, University of Genoa, IRCCS AOU San Martino - IST, Genova, Italy; 3 Department of Hearth, Environmental and Life Science, Center for Excellence in Biomedical Research, Genova, Italy; 4 Department of Experimental Medicine and Center for Excellence in Biomedical Research, Genova, Italy; 5 Sirius-biotech, c/o Advanced Biotechnology Center, Genova, Italy; 6 Protein Expression and Purification Core Facility, EMBL Heidelberg, Heidelberg, Germany; New England BioLabs, United States of America

## Abstract

*Escherichia coli* is a robust, economic and rapid expression system for the production of recombinant therapeutic proteins. However, the expression in bacterial systems of complex molecules such as antibodies and fusion proteins is still affected by several drawbacks. We have previously described a procedure based on uteroglobin (UG) for the engineering of very soluble and stable polyvalent and polyspecific fusion proteins in mammalian cells (Ventura et al. 2009. J. Biol. Chem. 284∶26646–26654.) Here, we applied the UG platform to achieve the expression in *E. coli* of a bivalent human recombinant antibody (L19) toward the oncofetal fibronectin (B-FN), a pan-tumor target. Purified bacterial L19-UG was highly soluble, stable, and, in all molecules, the L19 moiety maintained its immunoreactivity. About 50–70% of the molecules were covalent homodimer, however after refolding with the redox couple reduced-glutathione/oxidized-glutathione (GSH/GSSG), 100% of molecules were covalent dimers. Mass spectrometry studies showed that the proteins produced by *E. coli* and mammalian cells have an identical molecular mass and that both proteins are not glycosylated. L19-UG from bacteria can be freeze-dried without any loss of protein and immunoreactivity. *In vivo,* in tumor-bearing mice, radio-iodinated L19-UG selectively accumulated in neoplastic tissues showing the same performance of L19-UG from mammalian cells. The UG-platform may represent a general procedure for production of various biological therapeutics in *E. coli.*

## Introduction

Fibronectins (FNs) are high molecular mass adhesive glycoproteins of the extracellular matrix (ECM). FNs are widely distributed in normal tissues and body fluids and are involved in several processes such as cell adhesion and migration, maintenance of normal cell morphology, cell growth and differentiation [1], [2]. Fibronectins are encoded by a single gene localized on chromosome 2 [3], but different isoforms arise from the alternative splicing of the pre-mRNA, a process that for some ECM proteins, including fibronectin, is modulated by cytokines and extracellular/intracellular pH [4]–[6].

B-FN is a FN isoform containing the extra-domain B (ED-B), a complete type III homology repeat of 91 amino acids in which exon usage or skipping leads to inclusion or exclusion within the molecule of this type III repeat. B-FN is undetectable in tissues of healthy adults (with very rare exceptions, such as the female reproductive system where recurrent tissue remodeling and angiogenesis processes take place), it is abundant in fetal tissues and in different types of pathologies including cancer and all angiogenesis-associated pathologies [7]–[12]. The demonstration that murine monoclonal antibodies to B-FN injected into tumor-bearing mice selectively accumulate in neoplastic lesions [13] prompted the generation of high-affinity human recombinant antibodies for therapeutic and diagnostic purposes [14]–[21].

L19 is a human high-affinity recombinant antibody that reacts with the ED-B domain [15]. Because the ED-B domain shares 100% homology in human and mouse, L19 recognizes both human and murine B-FN. Several studies have demonstrated that L19 can be used to selectively deliver radionuclides or toxic agents to tumors both in diagnostic and therapeutic applications. L19 is presently used in phase I/II clinical trials for therapy of both solid and hematologic malignancies as a radio-immunoconjugate or as a fusion protein with the cytokines TNF-alpha and IL-2 [17], [22]–[25].

L19 has been produced in various formats including dimeric scFv (scFv2) in *E. coli*, small immunoprotein (SIP) and complete IgG1 in mammalian cells, and these formats show diverse pharmacokinetic properties [19]. SIP, produced in CHO cells, is the currently used format for radioimmuno-therapeutic purposes because it represents the best compromise of *in vivo* stability, blood clearance and performance in tumor targeting [19]. In particular the performance in tumor targeting of L19 scFv was very poor since it was unstable giving formation of aggregates and losing its immunoreactivity few hours after injection [19].

**Figure 1:**
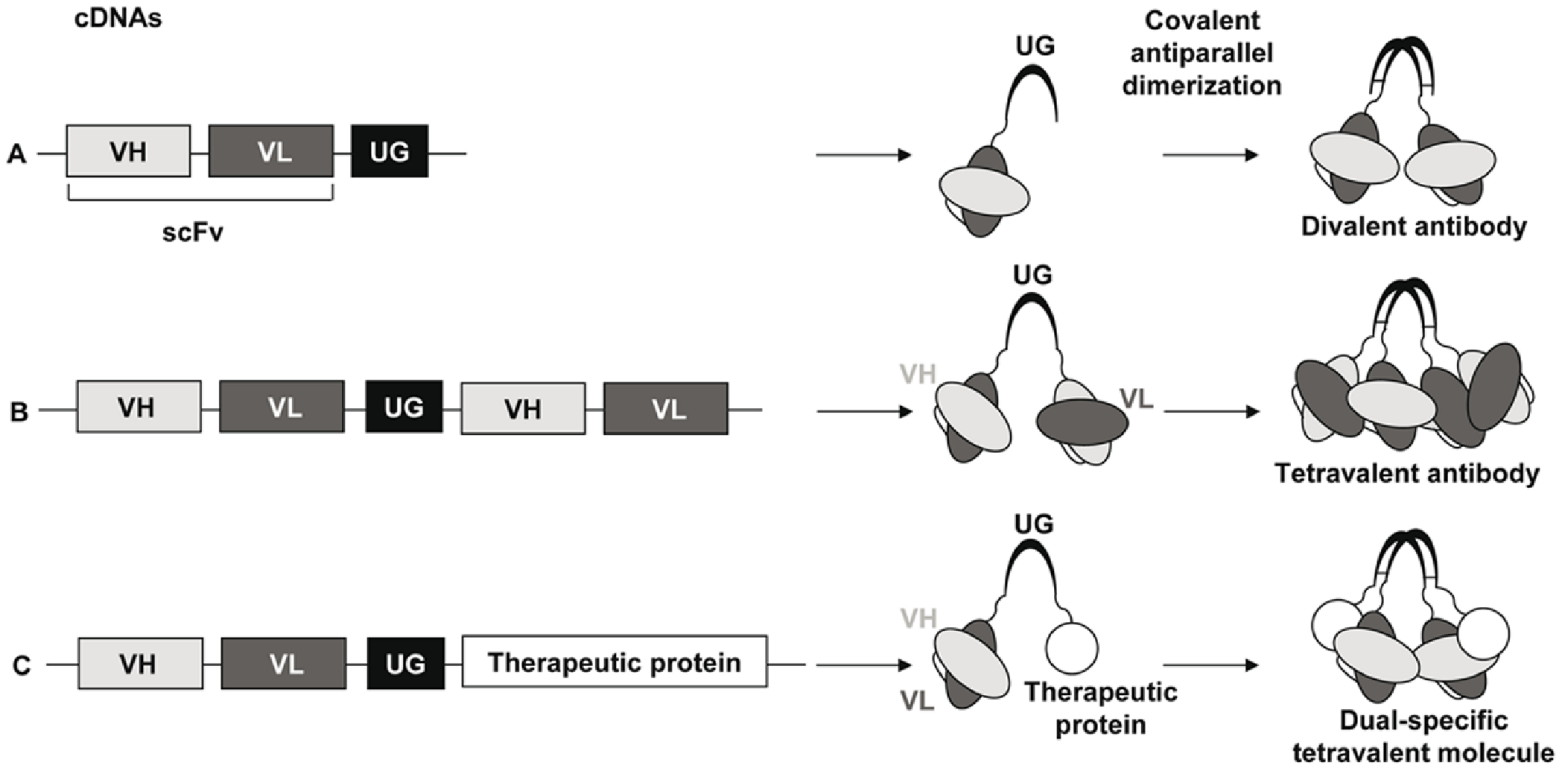
Uteroglobin platform. A) The cDNA encoding the variable heavy (VH) and variable light (VL) domains of immunoglobulins composing a single chain fragment variable (scFv) is fused to the 5′ end of uteroglobin (UG) cDNA. The covalent dimerization of the UG moiety within the fusion protein scFv-UG allows the generation of a divalent antibody. B) The cDNA encoding for an scFv is fused to both the 5′ and 3′ ends of UG. The resulting fusion protein is a tetravalent antibody. C) Two different cDNAs encoding for two different antibody fragments or an antibody fragment and a therapeutic molecule, such as a cytokine, are fused to the 5′ and 3′ ends of UG. The resulting protein is dual specific and tetravalent [26].

We recently described a novel strategy for the generation of divalent and dual-specific tetravalent antibodies based on the use of uteroglobin (UG) [26], [27]. UG is a seventy-amino acids globular and non-glycosylated homodimeric secreted protein [28]. The UG monomer is organized into a secondary structure containing four alpha helices; two subunits are then joined in an antiparallel fashion by disulfide bridges established between two highly conserved cysteine residues in the amino and carboxyl termini [28]. The high solubility and stability of UG to variations in pH and temperature, its resistance to proteases and its homodimeric structure make UG an ideal linker for the generation of polyvalent and either monospecific or bispecific recombinant antibodies. The UG platform (Fig. 1) consists of the fusion of the recombinant antibody sequence at the amino terminal or alternatively at the carboxyl terminal or both the amino and carboxyl terminals of UG; the covalent dimerization of UG allows the dimerization of the fusion proteins and thus the generation of divalent or dual specific-tetravalent molecules, which, when compared with similar fusion proteins without UG, possess enhanced solubility and stability, factors that would improve their storage and clinical use [26]. L19-UG is very soluble and stable and has a better performance with respect to the SIP for *in vivo* accumulation in neoplastic tissues in tumor-bearing mice [26]. However, until now, both the SIP and UG formats of L19 have been produced in mammalian cells. Their expression and purification from bacteria would be beneficial because the production of recombinant therapeutic proteins from *E. coli* offers several advantages over mammalian cells including higher yields, faster and simpler growth, lower costs and easier scale up processes [29]. In fact, numerous efforts have been made to produce complex molecules in bacteria, in particular a procedure for isolating full-length antibodies from libraries expressed in *E. coli* has been described [30].

Here we report the expression, purification and characterization both *in vitro* and *in vivo* of L19-UG from *E. coli* demonstrating the possibility of using the UG platform for the production of complex therapeutic fusion proteins in bacterial systems.

## Materials and Methods

All experiments involving animals were reviewed and approved by the Ethical Committee of the National Cancer research Institute’s Animal Facility and in compliance with the current National and International guidelines of FELASA, and designated by the Italian Ministry of Health with Ministerial Decree D.M.S. n° 146/2009-A and subsequent integration, project n° 282.

### L19-UG cDNA Construct and Protein Expression

The cDNA sequence encoding the scFv L19 protein was provided by Shine Gene Molecular Biotech (Shanghai, China) and the cDNA sequence encoding the human fusion protein L19-UG, which was optimized for expression in *E. coli* and cloned into the pUC57 vector, was provided by GenScript (Piscataway, NJ). The cDNA sequence was amplified by PCR as previously described [26] using the forward primer 5′- ctcccatggccgaagttcagctgctggaaagc-3′, containing the NcoI site, and the reverse primer 5′-ctcgcggccgcttagttgcacaggctgct-3′, containing a stop codon and the NotI site. The cDNA of L19-UG and the pHEN-1 expression vector [31] were both digested by NcoI/NotI, ligated and used to transform DH5α bacteria as previously described [26]. The cDNA construct was extracted and purified from DH5α, sequenced on both strands and used to transform the TG-1 and HB2151 *E. coli* strains.

L19-UG was expressed in *E. coli* HB2151 or TG-1 strains transformed with the pHEN-1 vector containing the cDNA construct of L19-UG. Bacteria was grown at 37°C with shaking in 2xYT broth (MP Biomedical, Santa Ana, CA) supplemented with 1% D-glucose and 100 µg/ml ampicillin (Sigma, St. Louis, MO). This overnight culture was diluted 1∶100 to prepare a culture in 2xYT broth supplemented with 0.1% D-glucose and 100 µg/ml ampicillin. Bacteria were incubated at 37°C until the culture reached an A_600_ value of 0.6, at which point it was incubated at 18°C for 30 minutes. Expression of the protein was then induced by adding isopropyl-β-D-thiogalactopyranoside (IPTG) (Inalco, Italy) at a final concentration of 0.2 mM and by incubating at 18°C for 72 hours. Triton X-100 (Sigma) was also added at the final concentration of 1% (v/v).

### Immunoaffinity, Anion Exchange and Size Exclusion Chromatography

After removing bacterial cells by centrifugation at 5200×*g* for 1 hour at 4°C using a Avanti J-25 centrifuge (Beckman Coulter, Brea, CA), the bacteria culture broth was first passed on a pre-column of Sepharose-4B and then loaded on an immunoaffinity chromatography column of recombinant ED-B conjugated to Sepharose-4B (GE Healthcare, Waukesha, WI). The column was washed with 3 column volumes of phosphate buffered saline (PBS, 20 mM NaH_2_PO_4_, 150 mM NaCl, pH = 7.6) followed by 2 column volumes of 20 mM NaH_2_PO_4_ (pH = 7.6) containing 1 M NaCl and then 2 column volumes of PBS. The protein was then eluted in 10 mM tri-ethilamine (pH = 11.0) and dialyzed in a solution containing 20 mM Tris/HCl and 28 mM NaCl (pH = 8.2).

Anion exchange chromatography was conducted using a HiTrap DEAE FF column (GE Healthcare) connected to an AKTA Basic system (GE Healthcare). L19-UG in a solution of 20 mM Tris/HCl and 28 mM NaCl (pH = 8.2) was loaded on the column, which was previously equilibrated with the same buffer, and then eluted with a solution containing 20 mM Tris/HCl and 90 mM NaCl (pH = 8.2).

Size exclusion chromatography (SEC) was carried out using a Superdex200 column (GE Healthcare) equilibrated in PBS (pH = 7.6) and the AKTA Basic System.

### Protein Refolding

Protein refolding was performed by adding to the protein in solution in PBS, 1 mM GSH and 0. 2 mM GSSG (final pH ∼ 8) and by incubating at 4°C for 24 hours. Refolded L19-UG was than separated from glutathione by SEC on a Superdex 200 column, equilibrated in PBS.

### Sodium Dodecyl Sulfate-polyacrylamide Gel Electrophoresis (SDS-PAGE), Enzyme-linked Immunosorbent Assay (ELISA) and Recombinant FN Fragments

4–12% SDS-PAGE gradient analysis in reducing and non-reducing conditions was carried out as previously described [19]. The 7.ED-B.8.9, ED-B and B-8 recombinant fragments of FN were prepared as described previously [32].

ELISA was performed as previously described [32]. Briefly, different concentrations of purified protein were tested against the fibronectin recombinant fragment 7.ED-B.8.9. Bound L19-UG was revealed by using a rabbit polyclonal antibody to human uteroglobin (produced in our laboratory). A peroxidase-conjugated anti-rabbit IgG was used as the tertiary antibody (Pierce, Rockford, IL).

### Mass Spectrometry

The molecular mass of L19-UG was measured using an Agilent 1100 HPLC system coupled to a MSD Ion Trap XCT mass spectrometer, equipped with an electrospray ion source (HPLC-ESI-MS) (Agilent Technologies, Palo Alto, CA, USA). To evaluate the presence of essential disulfide bridges the samples were analyzed in the presence or in the absence of the disulfide reducing agent dithiothreitol (DTT). The reduction was conducted with 150 mM DTT at room temperature for 5 hours. Separations were performed on a Symmetry C_4_ column 1×150 mm with 3-µm particle size (Waters Corporation, Milford, MA, USA).

Eluents used were water and acetonitrile added with 0.1% formic acid. The gradient employed was: 10% acetonitrile for 10 min, then linear to 95% in 50 min. The flow rate was set to 30 µl/min and the column temperature was set at 25°C. Injection volume was 5 µl. Ions were detected in ion charged control with a target ions value of 50000 and an accumulation time of 300 ms, using the following operation parameters: capillary voltage: 2500V; nebulizer pressure: 25 psi; drying gas: 8 l/min; dry temperature: 325°C; rolling averages 3, averages 8. Mass spectra were acquired in the positive ion mode in the 800–2000 m/z mass range. Raw spectra were deconvoluted using the LC/MSD Trap Software revision 5.3.

### Cell Lines, Immunohistochemistry (IHC) and Immunofluorescence (IF)

Human melanoma derived cells SK-MEL-28, human SV40 transformed WI38VA fibroblasts and mouse embryonic teratocarcinoma cells F9 were purchased from ATCC (Rockville, MD) and cultured in DMEM (Sigma) supplemented with 10% FCS (Sigma) and 4 mM L-glutamine (Biochrom, Berlin, Germany). For IF studies, cells were fixed with ice cold (−20°C) acetone (Sigma).

For immunohistochemical and immunofluorescence experiments, we used cultured human SV40 transformed WI38VA fibroblasts and 5-µm cryostat sections of the human melanoma SK-MEL-28 subcutaneously grown in NOD-SCID mice. IHC was performed as previously described [10] using biotinylated L19-UG at a final concentration of 2 µg/ml. For L19-UG biotinylation, we used the reagent 2X-AH-BIOTIN-NHS (Biospa, Milano, Italy) following the manufacturer’s instructions. For IF we used L19-UG at a concentration of 1 µg/ml and a rabbit polyclonal anti-human uteroglobin antibody (Abcam, Cambridge, UK) as the secondary antibody. A Cy3-conjugated goat anti-rabbit IgG (H+L) antibody (Jackson ImmunoResearch, West Grove, PA) was used as the tertiary antibody. The sections were counterstained with DAPI (Invitrogen, Carlsbad, CA). Images were acquired using a Nikon Digital Sight DS-5Mc camera mounted on an Olympus BX5 I microscope and the Nikon imaging software NIS-Elements F.

### Radio-labelling of L19-UG with ^125^I and Biodistribution Experiments

L19-UG was radio-labeled with ^125^I by using the IODO-GEN method (Pierce, Rockford, IL) as previously described [19]. The immunoreactivity of the radiolabeled protein was determined as previously reported [19]. ^125^I –L19-UG was analyzed by SEC, by loading about 10 µCi of radio-labeled protein on a Superdex 200 column equilibrated in PBS and counting the eluted fractions with a gamma-counter (Cobra, Packard/Perkin Elmer, Waltham, USA). The radiochemical purity (RCP) of ^125^I –L19-UG was assessed by Instant Thin Layer Chromatography (ITLC). A 20 cm long ITLC SG (Gelman Sciences, Ann Arbor, MI) and 85% methanol in water were used as stationary and mobile phases, respectively. After a run of 15 cm, the stationary phase was used to expose a super resolution Type SR autoradiography plate (Packard/PerkinElmer) for 30 minutes. Data were acquired by using Cyclon (Perkin Elmer).

For biodistribution experiments male NOD-SCID mice were subcutaneously implanted with 3,5×10^6^ F9 cells in 100 µl of PBS. When tumors reached the volume of approximately 0.1–0.3 cm^3^ (determined using the formula (d)^2^ × D × 0.52 where d and D are the short and long dimensions respectively, determined using a calliper), animals were treated intravenously with 4,5 µCi of ^125^I –L19-UG. To block nonspecific accumulation of ^125^I in the stomach and concentration in thyroid, mice were given orally 20 mg of sodium perchlorate (Carlo Erba, Milano, Italy) in water, 30 minutes before injection of the radio-labeled antibody. Animals were dived into three groups of three mice each and sacrificed 3 hours and 30 minutes, 24 and 48 hours post injection. Tumors and organs were excised, weighed and counted in a gamma counter (Perkin Elmer). The accumulation of the radio-labeled protein in the different organs is expressed as the percentage of the injected dose per gram of tissue (%ID/g).

NOD-SCID mice were provided by the IRCCS AOU San Martino – IST Animal Facility (Genova, Italy). All procedures involving animals were performed under the supervision and approval of the Ethical Committee of the National Cancer Research Institute’s Animal Facility and in compliance with the current national and international guidelines of FELASA, and designated by the Italian Ministry of Health with Ministerial Decree D.M.S. n° 146/2009-A and subsequent integrations, project n° 282.

## Results

### Protein Expression, Purification, Characterization and Refolding

**Figure 2:**
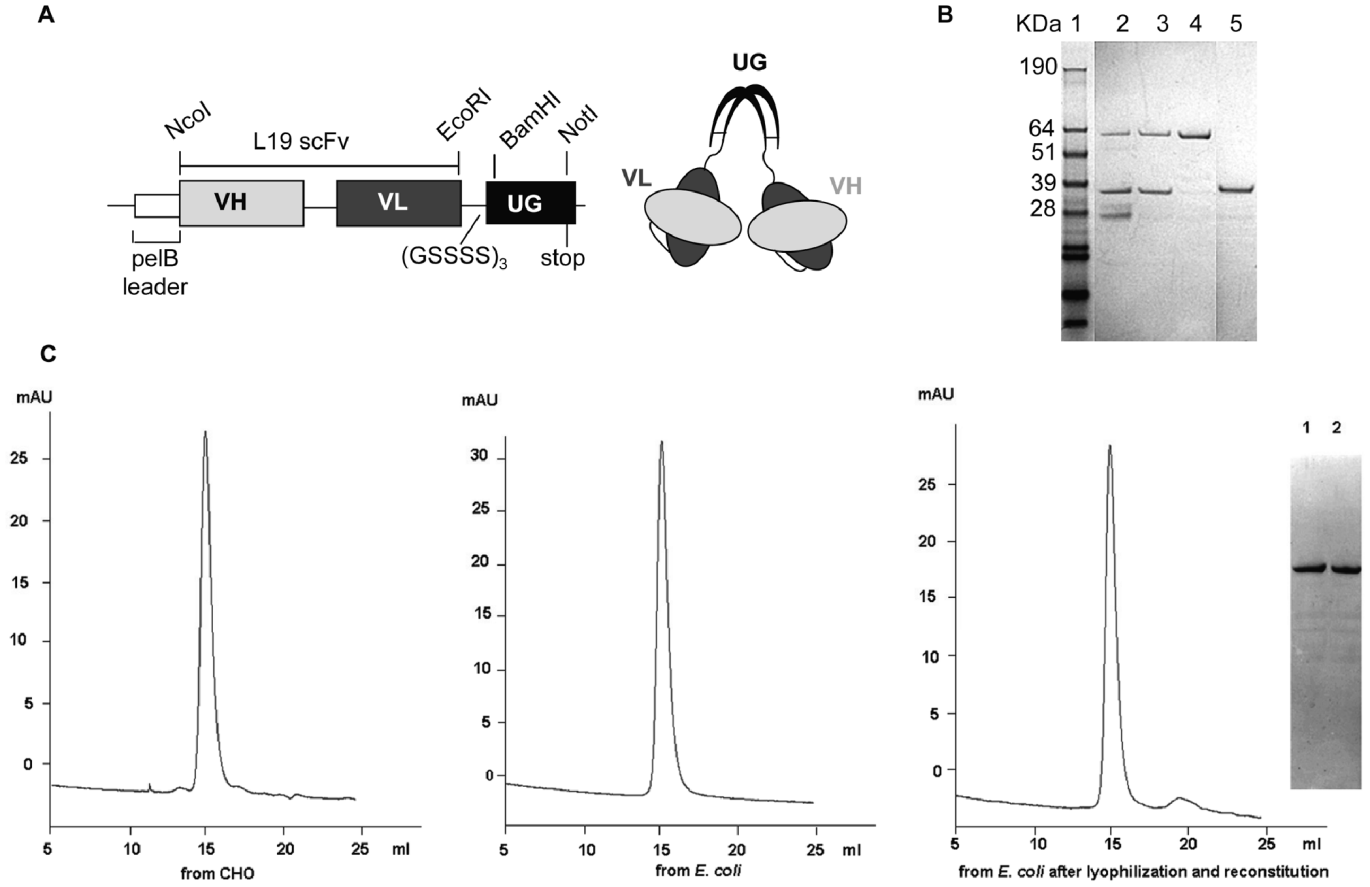
L19-UG from *E. coli*. **A)** Schematic of the L19-UG cDNA cloned into the pHEN-1 prokaryotic expression vector and fused to the pelB leader sequence. Depicted on the right is a schematic representation of the resulting dimeric fusion protein. **B)** SDS-PAGE analysis of L19-UG under non-reducing conditions after the first (lane 2) and the second purification steps (lane 3). L19-UG after protein refolding under non-reducing (lane 4) and reducing conditions (lane 5). Lane 1 shows the molecular mass standards. **C)** Size-exclusion chromatography (Superdex200 column) profiles of L19-UG purified from CHO cells (left panel) and from *E. coli* (central panel). The column retention volumes are 15.18 ml for the protein purified from CHO cells and 15.14 ml for the protein purified from *E. coli.* In the right panel the SEC profile of L19-UG obtained from *E. coli* after protein lyophilization and reconstitution. On the right the SDS-PAGE analysis of L19-UG, under non-reducing conditions, before (lane 1) and after (lane 2) protein lyophilization and reconstitution.

For the expression of L19-UG, we used the prokaryotic expression vector pHEN-1, which includes the bacterial PelB signal sequence for protein export into the periplasm (Fig. 2A). We used the HB2151 and TG-1 *E. coli* strains as expression host strains and obtained similar yield of L19-UG with similar solubility, stability and immunoreactivity from both strains. We cultivated bacteria under different temperatures (from 30°C to 10°C), inducer concentrations (from 0.01 to 1 mM), incubation times (from 16 to 72 hours) and additive amounts. We found that the best conditions for L19-UG expression were as follows: 0.2 mM IPTG, induction temperature of 18°C, incubation time of 72 hours and supplementation with 1% (v/v) Triton-X100. Under these conditions, the release of L19-UG from the periplasm into the culture broth was higher than 95%, while without Triton-X100 the release from the periplasm into the culture media was about 50%.

We purified L19-UG from the bacteria culture broth in two steps. In the first step, the protein was purified by immunoaffinity chromatography with ED-B conjugated to Sepharose-4B as described in “Materials and Methods.” This purification produced a yield of approximately 5–6 mg of protein per liter of bacteria culture broth. L19-UG was than extensively dialyzed against a solution of 20 mM Tris/HCl and 28 mM NaCl (pH = 8.2). Fig. 2B shows the SDS-PAGE of L19-UG obtained after the first purification step. Under non-reducing conditions, L19-UG migrates both as a monomer and a dimer with apparent molecular mass of about 35 kDa and 64 kDa, respectively. Since different contaminating bands of lower molecular mass were also present, L19-UG was then loaded on an anion exchange chromatography HiTrap DEAE FF resin that was previously equilibrated in a solution of 20 mM Tris/HCl and 28 mM NaCl (pH = 8.2). Under these conditions, contaminants did not bind to the resin, while L19-UG was retained by the resin. The protein was than eluted using 90 mM NaCl in 20 mM Tris/HCl (pH = 8.2). As shown by the SDS-PAGE analysis (Fig. 2B), this second step of purification allowed the separation of L19-UG from contaminants.

Since purified L19-UG from *E. coli* was only 50–70% a covalent dimer, we treated the protein with the redox couple GSH/GSSG to achieve 100% covalent dimerization. As shown in Fig. 2B, after incubation at 4°C for 24 hours in presence of 1 mM GSH and 0.2 mM GSSG, L19-UG became 100% covalent dimer, migrating in SDS-PAGE under non-reducing conditions as a single band with an apparent molecular mass of 64 KDa. In reducing condition the protein migrated as a single band with an apparent molecular mass of about 35 KDa, as expected (Fig. 2B). Identical bands under reducing and non-reducing conditions were observed for L19-UG purified from CHO cells [26]. SEC of the purified and refolded L19-UG shows a single peak with a retention volume of approximately 15 ml, which is in accordance with the molecular mass of dimeric L19-UG and which coincides with the retention volume obtained with the L19-UG purified from CHO cells (Fig. 2C).

L19-UG, in solution in PBS, was lyophilized and then reconstituted with distilled water without any protein loss. As demonstrated by the SEC and SDS-PAGE analysis, neither protein aggregation nor degradation were observed after lyophilization and reconstitution (Fig. 2C). On the contrary L19-SIP, as well as the L19-scFv from *E. coli*, has much lower solubility than L19-UG from *E.coli* and can not be reconstituted after lyophilization without aggregation and precipitation of protein [26].

**Figure 3:**
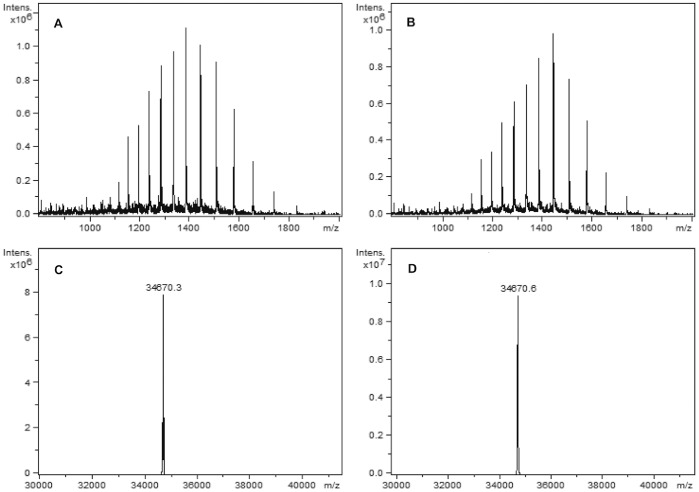
Mass spectrometry analysis of L19-UG from mammalian cells and *E.coli.* Mass spectrometry analysis of reduced L19-UG obtained from CHO cells (**A**, **C**) and from *E. coli* (**B**, **D**). The raw (**A**–**B**) and deconvoluted (**C**–**D**) mass spectra relative to the chromatographic peaks at 32.5 minutes are reported. The calculated average neutral mass of monomeric form of L19-UG is 34670,3 Da for the protein produced in CHO cells and 34670,6 Da for the protein produced in *E. coli*.

The molecular mass of reduced L19-UG measured by mass spectrometry was of 34670.6 Da for L19-UG from *E. coli* and of 34670.3 Da for L19-UG from CHO cells (Fig. 3). The measured molecular mass coincides with the theoretical one, 34670.6 Da, calculated on the basis of aminoacid composition. The MS data indicate that: 1. the leader peptide is correctly removed from the fusion protein produced in *E. coli* during the secretion process and that no proteolytic degradation has occurred; 2) the protein obtained from CHO cells is not glycosylated.

**Figure 4:**
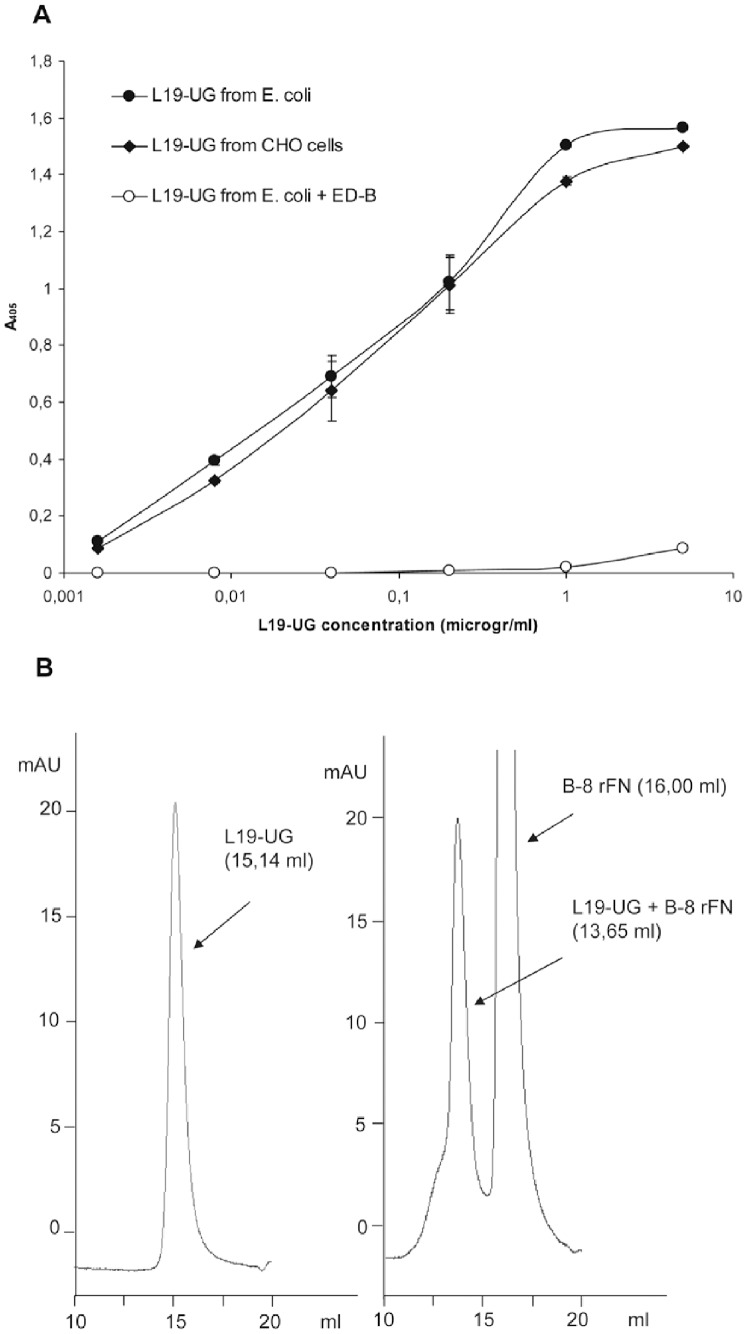
Immunoreactivity of L19-UG from *E.coli*. **A)** Different concentrations of L19-UG purified from *E. coli* (black circles) and CHO cells (black diamonds) were tested for binding to the recombinant fragment 7.ED-B.8.9 FN by ELISA. To verify the specificity of the binding, different concentrations of L19-UG from *E. coli* were pre-incubated with a molar excess (1,2 milligrams per ml) of the recombinant ED-B and then tested in ELISA for its binding to 7.ED-B.8.9 (white circles). The mean absorbances at λ = 405 nm ± SD are indicated. **B)** A shift in the column retention volume of L19-UG in SEC (Superdex 200) was obtained after incubating L19-UG with a molar excess of the recombinant FN fragment B-8. Left panel: SEC profile of L19-UG showing a single peak at 15.14 ml. Right panel: SEC profile of L19-UG pre-incubated with a molar excess of B-8 rFN showing an elution peak at 13.65 ml, which corresponds to the immunocomplex L19-UG/B-8 rFN, and an elution peak at 16 ml, which corresponds to the excess unbound B-8 rFN.

To test the immunoreactivity of the L19 moiety, different concentrations of L19-UG were used in ELISA against the human recombinant FN fragment containing the type III repeats 7, ED-B, 8 and 9 and compared with L19-UG that was purified from mammalian CHO cells. Fig. 4A shows that the proteins purified from *E. coli* and from CHO cells gave identical results, which demonstrates identical immunoreactivity and avidity of the L19 moiety in both proteins. The reaction was abolished by pre-incubating L19-UG with a large excess of purified ED-B, which further demonstrates the specificity of the reaction. To test the immunoreactivity in solution, we incubated L19-UG with a seven-fold molar excess of a recombinant FN fragments composed of ED-B and 8 type III repeats (B-8 rFN), and then analyzed them by SEC. The peak of L19-UG completely shifted from approximately 15 ml to approximately 13.7 ml (Fig. 4B), indicating a complete binding of L19-UG to the rFN fragment B-8, thus demonstrating that 100% of the molecules were immunoreactive. Identical results, both in ELISA and SEC, were obtained by testing L19-UG before the refolding step (data not shown). This demonstrates that the protein obtained from *E. coli*, even if not 100% covalently dimer, was 100% immunoreactive.

**Figure 5:**
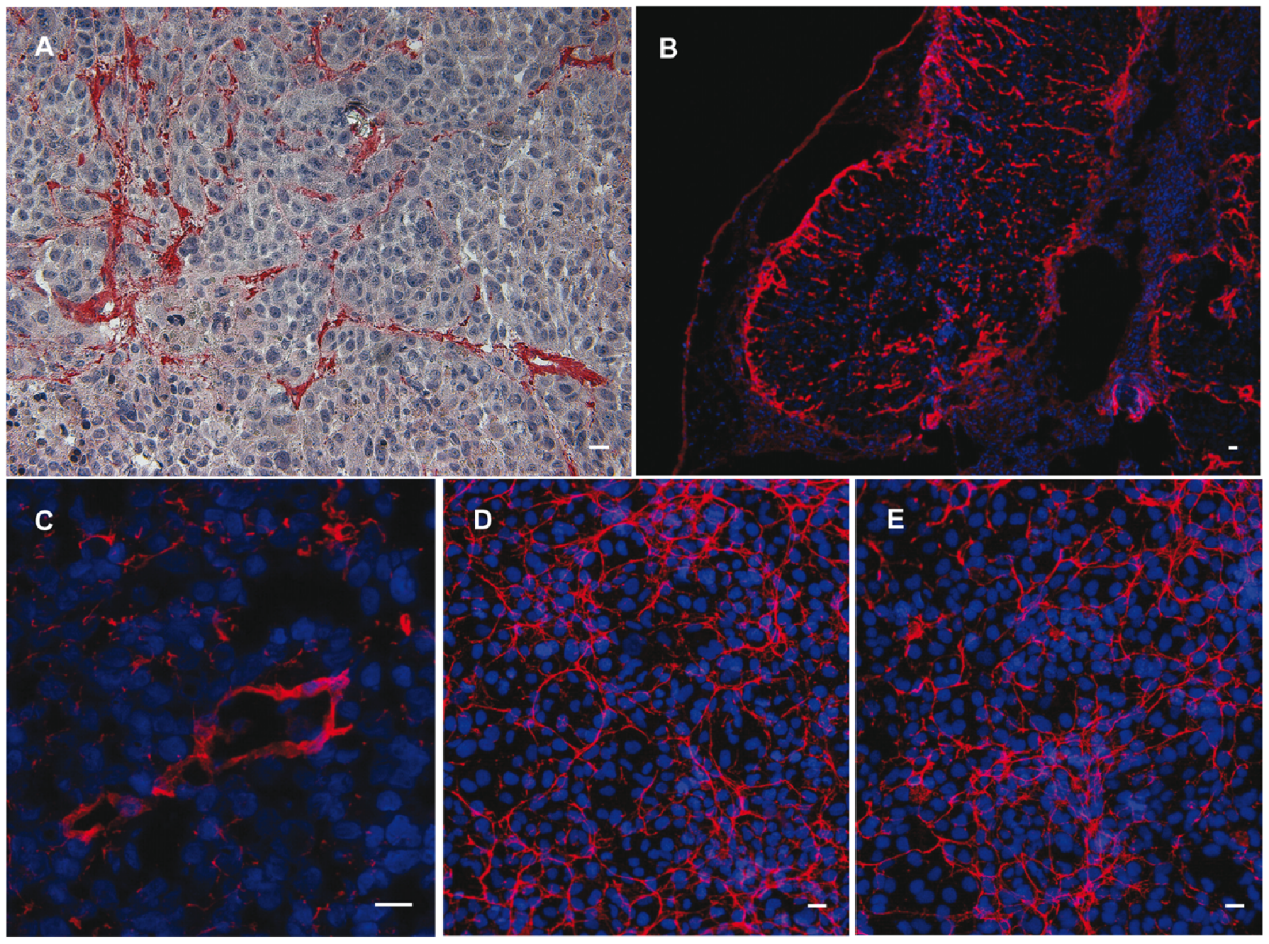
Immunohistochemistry and immunofluorescence experiments. **A)** IHC analysis of human melanoma SK-MEL-28 specimens grown subcutaneously in NOD-SCID mice. L19-UG purified from *E. coli* (red) detects tumor-associated vessels. **B–C)** IF analysis of SK-MEL-28 specimens: L19-UG from *E. coli* (red) detects fibrillar structures of the tumor extracellular matrix (**B**) and vessels (**C**)**. D–E)** IF analysis of cultured human SV40 transformed WI38VA fibroblasts stained with L19-UG purified from *E. coli* (**D**) and CHO cells (**E**)**.** L19-UG detects the typical FN fibrillar structures of the extracellular matrix. Nuclei were stained with DAPI. Bars: 5 µm.

L19-UG was used in immunohistochemistry and immunofluorescence on cultured transformed human fibroblasts (WI38VA) and specimens of human melanoma SK-MEL-28 grown subcutaneously in NOD-SCID mice. L19-UG detected tumoral vessels as expected (Fig. 5A–C). Equivalent amounts of L19-UG obtained from *E. coli* or CHO gave the same reaction patterns, typical of B-FN (Fig. 5D–E).

### Biodistribution in Tumor-bearing Mice

**Figure 6:**
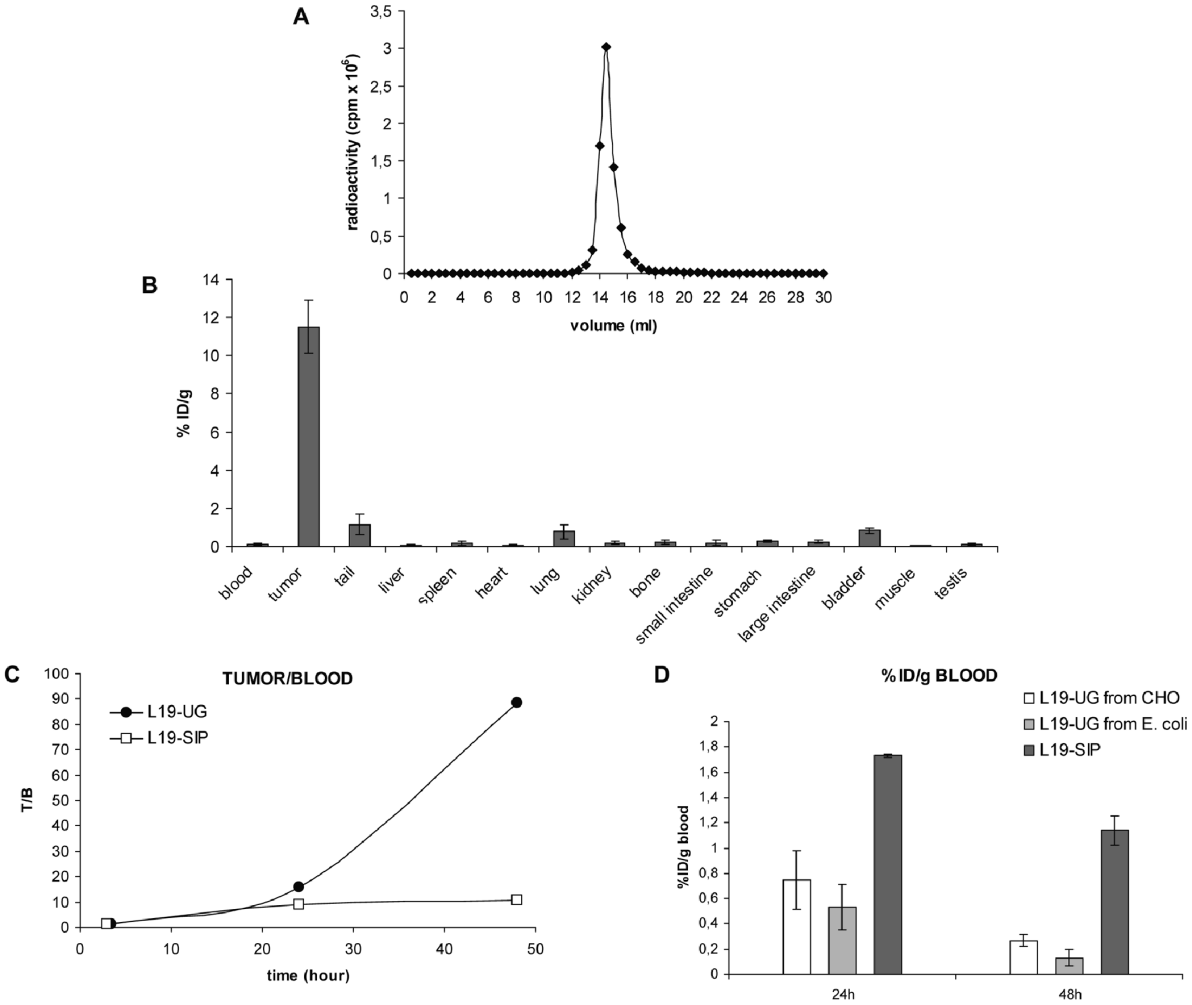
Bio-distribution of ^125^I-L19-UG in tumor bearing mice. **A**) SEC analysis of ^125^I-L19-UG (Superdex 200). The retention volume of about 14.5 ml is in accordance with the molecular mass of dimeric L19-UG. **B–D)** Biodistribution of ^125^I-L19-UG in F9 tumor-bearing mice. **B)** The percentage of injected dose per gram of tissue (%ID/g) in the various organs 48 hours post i.v. administration of ^125^I-L19-UG. The means ± S.E. are indicated. **C)** The ratios of the %ID/g in tumor (T) and blood (B) of L19–UG and of the currently used L19 format SIP (data from reference 19) presently used in clinical. **D)** %ID/g in the blood at 24 and 48 hours after radio-labelled proteins injections using L19-SIP (data from reference 19), L19-UG produced in mammalian cells (data from reference 26) and L19-UG from *E. coli*. %ID/g and standard errors are shown.

L19-UG was radio-labeled with Iodine 125. After radio-iodination L19-UG was 95% immunoreactive. The SEC analysis of ^125^I-L19-UG shows a single peak with a column retention volume (14,5 ml), which is in accordance with the molecular mass of L19-UG, and the absence of any aggregates and proteolytic fragments (Fig. 6A). The RCP, determined by ITLC, one hour after radiolabeling, was higher than 95%. ^125^I-L19-UG was tested in bioistribution experiments in F9 teratocarcinoma tumor-bearing mice. As shown in Fig. 6B–C and in Table 1, L19-UG selectively accumulated in the tumors, reaching a percentage of the injected dose per gram of tissue (%ID/g) of about 12% 48 hours post-injection (Fig. 6B and Table 1). The radio labeled molecule was cleared quickly from the blood, showing a %ID/g in the blood at 24 and 48 from injection of 0,53% and 0,13%, respectively (Fig. 6D and Table 1). Forty-eight hours post-injection the %ID/g in the tumor was 88 fold higher than the %ID/g in blood (T/B) while the T/B was about twelve employing the format currently in use in clinical trials, L19 SIP [19] (Fig. 6C and 6D).

**Table 1:** Biodistribution experiments of ^125^I-L19-UG in F9 tumor-bearing mice.

ORGAN	3 hr 30 min	24 hr	48 hr
Blood	11,17±2,39	0,53±0,31	0,13±0,11
Tumor	14,23±2,12	8,40±2,15	11,52±2,38
Liver	4,06±1,21	0,19±0,09	0,08±0,06
Spleen	4,57±1,79	0,29±0,17	0,16±0.20
Heart	4,57±1,6	0,22±0,12	0,07±0,06
Lung	7,92±0,72	0,64±0,31	0,78±0,66
Kidney	7,12±1,52	0,40±0,17	0,18±0,14
Bone	2,23±0,21	0,47±0,34	0,23±0,19
Stomach	1,80±0,23	0,61±0,34	0,27±0,25
Small intestine	3,04±0,60	0,63±0,10	0,18±0,07
Large intestine	2,05±0,71	0,48±0,21	0,25±0,13
Bladder	10,63±6,25	1,78±0,63	0, 83±0,23
Muscle	0,99±0,19	0,21±0,24	0,05±0,05
Testis	3,14±0,11	0,20±0,08	0,10±0,1

The percentage of the injected dose per gram of tissue (%ID/g) ± SD in the different organs, at the indicated time post ^125^I-L19-UG i.v. administration, are reported.

## Discussion

Recombinant antibodies represent one of the most prominent drugs for imaging and for therapy of cancer and immune disorders. They include antibodies targeting antigens on cancer cells as well as antigens associated with cancer ECM and vasculature. Antigens associated with the tumor ECM and vessels offer several advantages with respect to cell surface antigens because they usually are more homogenous, more abundant, more stable, more accessible from the blood stream and they are shared by different types of malignancies [33].

B-FN is an oncofetal FN isoform [7], [8] and is the prototype ECM target arising from the deregulation of alternative splicing. In fact, the altered alternative splicing of many different pre-mRNA in several types of neoplastic and non-neoplastic diseases is recognized as a general phenomenon in which the derived products are considered important therapeutic and diagnostic targets [34].

The demonstration that B-FN is a cancer- and angiogenesis-associated ECM component and that murine monoclonal antibodies against B-FN that are injected i.v. into tumor-bearing mice selectively accumulate on neoplastic tissues, prompted the production of human recombinant antibodies from human scFv libraries [14]. One of these human recombinant antibodies, L19 [15], is currently used by Philogen SPA for various diagnostic and therapeutic clinical trials as a radio-immunoconjugate or as an immunocytokine in different tumor types [17], [21]–[25]. For radio immunotherapy of hematologic cancers, L19 is used in the radio iodinated small immunoprotein (SIP) format [17], [22].

We previously described the UG platform that allows the generation of very soluble and stable recombinant polyvalent/polyspecific antibodies in mammalian cells [26]. Here we demonstrate that this procedure allows the production of the antibody L19 in the UG format in *E. coli*. The L19-UG molecule produced in *E. coli* is very soluble and stable and maintains identical immunoreactivity and avidity compared to L19-UG produced from mammalian cells. The mechanisms for which UG increases the solubility of the fusion proteins are under investigation. Further, we demonstrated that the molecules expressed by mammalian cells and by *E. coli* have identical molecular mass and are not glycosylated.

Only 50–70% of L19-UG molecules from *E. coli* is a covalent dimer, even if 100% of L19-UG molecules is immunoreactive. However treating the purified protein with the redox couple GSH/GSSG is possible to obtain 100% of covalent dimer. After refolding the protein obtained from *E. coli* is identical to the molecule produced in CHO cells.

In tumor-bearing mice L19-UG from *E. coli* selectively accumulated in neoplastic tissues and showed a fast blood clearance. The biodistribution properties of L19-UG reported here confirm the previously obtained results with the protein expressed in mammalian cells. L19-UG performs better *in vivo* than the currently used SIP format in terms of accumulation in neoplastic lesions and blood clearance. In fact, in the murine tumor model F9, forty-eight hours post-injection the %ID/g in the tumor was 88 fold higher than the %ID/g in blood while it was about twelve employing L19-SIP, the format currently in use in clinical trials [19] (Fig. 6C and 6D). We are presently investigating the reason why fusion proteins containing UG have a faster clearance with respect to proteins not containing UG. Fast blood clearance is a very important drug property for radio-immunotherapy applications as it reduces the radio-toxicity on non-target organs. Moreover UG has anti-inflammatory properties and this could be helpful to contrast some radio-immunotherapy side effects such as inflammation and fibrosis.

In addition we have demonstrated that it is possible to lyophilize and reconstitute the protein without any protein precipitation and aggregation. This property, which is not shared by the antibody L19 in the SIP and scFv formats, can facilitate the storage and consequently the potential clinical use of L19-UG. In fact L19-SIP, as well as L19-scFv, after lyophilization and reconstitution presents significant amount of aggregates and precipitates [26].

The procedure for the production of L19-UG described here presents many advantages with respect to the production from mammalian cells. In fact, in general, recombinant protein production in *E. coli* is faster and cheaper than in mammalian systems. The cost for bacterial cell medium is at least 90% lower than for mammalian cell medium. Fermentation takes 24 to 72 hours in bacteria versus 14 days to 3 weeks in CHO cells. Furthermore, we have engineered a cDNA construct and described a procedure that induces the release of a correctly folded, soluble, and active recombinant protein directly into the extracellular fermentation broth, which allows simple and easily scalable GMP purification procedures. L19-scFv has been produced in *E. coli* and after purification it was made up of tow forms, monomer and a non-covalent dimer, a second step of purification was required to isolate the latter dimeric form [19]. When radio-labeled and tested *in vivo* in F9 tumor bearing mice the performance in tumor targeting of L19-scFv was very low, 3.2 and 2.8%ID/g at 24 and 48 hours after injection respectively, about three-four times lower with respect to the %ID/g obtained using L19-UG produced in *E. coli* [19]. Furthermore Borsi et al. reported that L19-scFv was unstable giving formation of aggregates and quickly loses its immunoreactivity few hours after injection in tumor-bearing mice [19].

In conclusion, the two main points reported here are 1) the possibility of utilizing bacteria to produce dimeric recombinant antibodies for tumor targeting and 2) that the UG platform could be useful for the production of complex therapeutic proteins in bacterial systems.

## References

[1] Hynes RO (1990) Fibronectins. New York: Springer-Verlag.

[2] Pankov R, Yamada KM (2002) Fibronectin at a glance. J Cell Sci 115: 3861–3863. 12244123 10.1242/jcs.00059

[3] Zardi L, Cianfriglia M, Balza E, Carnemolla B, Siri A, et al (1982) Species-specific monoclonal antibodies in the assignment of the gene for human fibronectin to chromosome 2. EMBO J 1: 929–933. 7188364 10.1002/j.1460-2075.1982.tb01273.x PMC553137

[4] Balza E, Borsi L, Allemanni G, Zardi L (1988) Transforming growth factor beta regulates the levels of different fibronectin isoforms in normal human cultured fibroblasts. FEBS Lett 228: 42–44. 3422628 10.1016/0014-5793(88)80580-5

[5] Borsi L, Balza E, Gaggero B, Allemanni G, Zardi L (1995) The alternative splicing pattern of the tenascin-C pre-mRNA is controlled by the extracellular pH. J Biol Chem 270: 6243–6245. 7534307 10.1074/jbc.270.11.6243

[6] Borsi L, Balza E, Castellani P, Carnemolla B, Ponassi M, et al (1994) Cell-cycle dependent alternative splicing of the tenascin primary transcript. Cell Adhes Commun 1: 307–317. 7521758 10.3109/15419069409097262

[7] Zardi L, Carnemolla B, Siri A, Petersen TE, Paolella G, et al (1987) Transformed human cells produce a new fibronectin isoform by preferential alternative splicing of a previously unobserved exon. EMBO J 6: 2337–2342. 2822387 10.1002/j.1460-2075.1987.tb02509.x PMC553637

[8] Carnemolla B, Balza E, Siri A, Zardi L, Nicotra MR, et al (1989) A tumor-associated fibronectin isoform generated by alternative splicing of messenger RNA precursors. J Cell Biol 108: 1139–1148. 2646306 10.1083/jcb.108.3.1139 PMC2115391

[9] Carnemolla B, Leprini A, Allemanni G, Saginati M, Zardi L (1992) The inclusion of the type III repeat ED-B in the fibronectin molecule generates conformational modifications that unmask a cryptic sequence. J Biol Chem 267: 24689–24692. 1280266

[10] Castellani P, Viale G, Dorcaratto A, Nicolo G, Kaczmarek J, et al (1994) The fibronectin isoform containing the ED-B oncofetal domain: a marker of angiogenesis. Int J Cancer 59: 612–618. 7525495 10.1002/ijc.2910590507

[11] Kosmehl H, Berndt A, Katenkamp D (1996) Molecular variants of fibronectin and laminin: structure, physiological occurrence and histopathological aspects. Virchows Arch 429: 311–322. 8982375 10.1007/BF00198435

[12] Castellani P, Borsi L, Carnemolla B, Biro A, Dorcaratto A, et al (2002) Differentiation between high- and low-grade astrocytoma using a human recombinant antibody to the extra domain-B of fibronectin. Am J Pathol 161: 1695–1700. 12414516 10.1016/S0002-9440(10)64446-X PMC1850782

[13] Mariani G, Lasku A, Balza E, Gaggero B, Motta C, et al (1997) Tumor targeting potential of the monoclonal antibody BC-1 against oncofetal fibronectin in nude mice bearing human tumor implants. Cancer 80: 2378–2384. 9406686 10.1002/(sici)1097-0142(19971215)80:12+<2378::aid-cncr7>3.3.co;2-0

[14] Carnemolla B, Neri D, Castellani P, Leprini A, Neri G, et al (1996) Phage antibodies with pan-species recognition of the oncofoetal angiogenesis marker fibronectin ED-B domain. Int J Cancer 68: 397–405. 8903484 10.1002/(SICI)1097-0215(19961104)68:3<397::AID-IJC20>3.0.CO;2-4

[15] Pini A, Viti F, Santucci A, Carnemolla B, Zardi L, et al (1998) Design and use of a phage display library. Human antibodies with subnanomolar affinity against a marker of angiogenesis eluted from a two-dimensional gel. J Biol Chem 273: 21769–21776. 9705314 10.1074/jbc.273.34.21769

[16] Neri D, Bicknell R (2005) Tumor vascular targeting. Nat Rev Cancer 5: 436–446. 15928674 10.1038/nrc1627

[17] Sauer S, Erba PA, Petrini M, Menrad A, Giovannoni G, et al (2009) Expression of the oncofetal ED-B-containing fibronectin isoform in hematologic tumors enables ED-B-targeted 131I-L19SIP radioimmunotherapy in Hodgkin lymphoma patients. Blood 113: 2265–2274. 19131554 10.1182/blood-2008-06-160416

[18] Carnemolla B, Borsi L, Balza E, Castellani P, Meazza R, et al (2002) Enhancement of the antitumor properties of interleukin-2 by its targeted delivery to the tumor blood vessel extracellular matrix. Blood 99: 1659–1665. 11861281 10.1182/blood.v99.5.1659

[19] Borsi L, Balza E, Bestagno M, Castellani P, Carnemolla B, et al (2002) Selective targeting of tumoral vasculature: comparison of different formats of an antibody (L19) to the ED-B domain of fibronectin. Int J Cancer 102: 75–85. 12353237 10.1002/ijc.10662

[20] Borsi L, Balza E, Carnemolla B, Sassi F, Castellani P, et al (2003) Selective targeted delivery of TNFalpha to tumor blood vessels. Blood 102: 4384–4392. 12933583 10.1182/blood-2003-04-1039

[21] Santimaria M, Moscatelli G, Viale GL, Giovannoni L, Neri G, et al (2003) Immunoscintigraphic detection of the ED-B domain of fibronectin, a marker of angiogenesis, in patients with cancer. Clin Cancer Res 9: 571–579. 12576420

[22] Erba PA, Sollini M, Orciuolo E, Traino C, Petrini M, et al (2012) Radioimmunotherapy with radretumab in patients with relapsed hematologic malignancies. J Nucl Med 53: 922–927. 22577235 10.2967/jnumed.111.101006

[23] Papadia F, Basso V, Patuzzo R, Maurichi A, Di Florio A, et al (2013) Isolated limb perfusion with the tumor-targeting human monoclonal antibody-cytokine fusion protein L19-TNF plus melphalan and mild hyperthermia in patients with locally advanced extremity melanoma. Journal of surgical oncology 107: 173–179. 22674435 10.1002/jso.23168

[24] Johannsen M, Spitaleri G, Curigliano G, Roigas J, Weikert S, et al (2010) The tumor-targeting human L19-IL2 immunocytokine: preclinical safety studies, phase I clinical trial in patients with solid tumors and expansion into patients with advanced renal cell carcinoma. Eur J Cancer 46: 2926–2935. 20797845 10.1016/j.ejca.2010.07.033

[25] Eigentler TK, Weide B, de Braud F, Spitaleri G, Romanini A, et al (2011) A dose-escalation and signal-generating study of the immunocytokine L19-IL2 in combination with dacarbazine for the therapy of patients with metastatic melanoma. Clin Cancer Res 17: 7732–7742. 22028492 10.1158/1078-0432.CCR-11-1203

[26] Ventura E, Sassi F, Fossati S, Parodi A, Blalock W, et al (2009) Use of uteroglobin for the engineering of polyvalent, polyspecific fusion proteins. J Biol Chem 284: 26646–26654. 19632988 10.1074/jbc.M109.025924 PMC2785352

[27] Ventura E, Balza E, Borsi L, Tutolo G, Carnemolla B, et al (2011) Selective targeted delivery of the TNF-alpha receptor p75 and uteroglobin to the vasculature of inflamed tissues: a preliminary report. BMC biotechnology 11: 104. 22074550 10.1186/1472-6750-11-104 PMC3226451

[28] Mukherjee AB, Zhang Z, Chilton BS (2007) Uteroglobin: a steroid-inducible immunomodulatory protein that founded the Secretoglobin superfamily. Endocr Rev 28: 707–725. 17916741 10.1210/er.2007-0018

[29] Huang CJ, Lin H, Yang X (2012) Industrial production of recombinant therapeutics in Escherichia coli and its recent advancements. J Ind Microbiol Biotechnol 39: 383–399. 22252444 10.1007/s10295-011-1082-9

[30] Mazor Y, Van Blarcom T, Mabry R, Iverson BL, Georgiou G (2007) Isolation of engineered, full-length antibodies from libraries expressed in *Escherichia Coli* . Nat Biotechnol 25(5): 563–565. 17435747 10.1038/nbt1296

[31] Hoogenboom HR, Griffiths AD, Johnson KS, Chiswell DJ, Hudson P, et al (1991) Multi-subunit proteins on the surface of filamentous phage: methodologies for displaying antibody (Fab) heavy and light chains. Nucleic Acids Res 19: 4133–4137. 1908075 10.1093/nar/19.15.4133 PMC328552

[32] Balza E, Sassi F, Ventura E, Parodi A, Fossati S, et al (2009) A novel human fibronectin cryptic sequence unmasked by the insertion of the angiogenesis-associated extra type III domain B. Int J Cancer. 125: 751–758. 10.1002/ijc.24473 19479996

[33] Zardi L, Neri D (1998) Affinity reagents against tumor-associated extracellular molecules and newforming vessels. Advanced drug delivery reviews 31: 43–52. 10837617 10.1016/s0169-409x(97)00093-8

[34] Miura K, Fujibuchi W, Unno M (2012) Splice isoforms as therapeutic targets for colorectal cancer. Carcinogenesis 33: 2311–2319. 23118106 10.1093/carcin/bgs347

